# Knee and hip osteoarthritis increase the risk of cardiovascular disease: A national registry-based longitudinal cohort study

**DOI:** 10.1371/journal.pone.0321290

**Published:** 2025-04-15

**Authors:** Jeffrey J. Hébert, Sinem Saritas, Parisa Niloofar, Sanja Lazarova-Molnar, Kim Christian Houlind, Niels Wedderkopp

**Affiliations:** 1 Faculty of Kinesiology, University of New Brunswick, Canada; 2 School of Allied Health, Murdoch University, Australia; 3 Department of Regional Health Research, University of Southern Denmark, Denmark; 4 Department of Vascular Surgery, Lillebaelt Hospital, Denmark; 5 Institute of Applied Informatics and Formal Description Methods, Karlsruhe Institute of Technology, Germany; 6 Maersk Mc-Kinney Moller Institute, University of Southern Denmark, Denmark; 7 Department of Clinical Research, University of Southern Denmark, Denmark; 8 The Orthopedic Department, Hospital of Southwestern Jutland, Denmark; University of Liege: Universite de Liege, Belgium

## Abstract

**Objective:**

Osteoarthritis and cardiovascular disease are major public health challenges. We aimed to estimate the average sex-specific effects of knee and hip osteoarthritis on the risk of cardiovascular disease.

**Methods:**

We used 2001–2015 Danish national health registry data to identify all adults with knee or hip osteoarthritis and an age-, sex-, and education-matched group without osteoarthritis. Cardiovascular disease outcomes were identified with relevant ICD-10 codes. The effects of osteoarthritis were estimated with sex-stratified multivariable Cox regression models, accounting for multiple sources of confounding determined a priori with a directed acyclic graph. Results were reported with cumulative incidence curves and hazard ratios (HR) conditioned on age, sex, education, and obesity diagnosis. Sensitivity analyses explored the potential impacts of bias owing to outcome misclassification and unmeasured confounding.

**Results:**

We analysed data from 1,838,434 adults, including 290,781 people with knee or hip osteoarthritis and 1,547,653 age-, sex-, and education-matched controls. Women with knee or hip osteoarthritis had a 44% increased hazard of cardiovascular disease (HR [95% CI] =  1.44 [1.43 to 1.46]), while men with knee or hip osteoarthritis had a 24% increased hazard of subsequent cardiovascular disease (HR[95% CI] =  1.24 [1.23 to 1.26]) compared to people without osteoarthritis. These results were confirmed by sensitivity analyses.

**Conclusion:**

The apparent effect of osteoarthritis on cardiovascular disease was stronger in women than in men. Clinicians who care for patients with osteoarthritis should be aware of cardiovascular disease risk when selecting therapies and consider behavioural approaches to improving health-related physical activity behaviour in this population.

## Introduction

Osteoarthritis and cardiovascular disease are major public health challenges. Worldwide, osteoarthritis is a leading source of disability, while heart disease is the number one cause of mortality.[1] Clinicians and researchers often consider osteoarthritis and cardiovascular disease in isolation despite the fact that nearly all people living with osteoarthritis have one or more co-morbid conditions, including 80% with cardiovascular disease.[2]

Multimorbidity (i.e., the coexistence of two or more long-term conditions)[3] negatively affects healthcare utilisation,[4] quality of life,[5] and mortality risk.[6] Osteoarthritis is an impactful risk factor for multimorbidity.[7] When musculoskeletal conditions are a part of the multimorbid state, they negatively impact physical health and healthcare costs.[8] Specifically, evidence points to osteoarthritis as a risk factor for cardiovascular disease and cardiovascular-related mortality.[9] Potential mechanisms explaining this link include pain-related reductions in physical activity,[10] increased use of non-steroidal anti-inflammatory drugs (NSAIDs),[11, 12] and persistent low-grade inflammation.[13] However, a recent systematic review[14] reported that prior studies have not sufficiently addressed confounding, and few studies have focussed on the sex-specific associations between osteoarthritis and cardiovascular disease.

Addressing the impact of sex and gender in research is a matter of social justice with tangible benefits, such as developing more effective health policies.[15] Unfortunately, sex and gender are often overlooked in the conduct and reporting of health research.[16] This reality has stimulated recent calls to integrate sex and gender in health research,[17] including in the fields of rheumatology[18] and orthopaedics.[19] Despite differences in the prevalence of osteoarthritis[20] and cardiovascular disease[21] between women and men, little evidence is available on the sex-specific effects of osteoarthritis on cardiovascular disease. Therefore, we aimed to estimate the average effects of knee and hip osteoarthritis on the risk of subsequent cardiovascular disease in women and men using data from a national health registry.

## Methods

### Study design and setting

We undertook a longitudinal study using 2001–2015 data from the Danish National Patient Register. Established in 1977, the Register contains prospectively collected inpatient and outpatient hospital health data for all Danish residents.[22] The international classification of disease version 10 (ICD-10) codes in the Danish National Patient Registry are valid compared to results from medical record reviews for both orthopaedic-related[23] and cardiovascular disease[24] diagnoses. This was a dynamic cohort in that participants initially assigned to the unexposed group, who later received a diagnosis of osteoarthritis, were subsequently classified as exposed from the date of diagnosis, and a new set of matched participants were added to the unexposed group. Participants were enrolled from 1 January 2001 to 31 December 2010 until the earliest of the following events: the outcome of interest, death, or 31 December 2015. It was not possible to extend the study period later than this date owing to changes in the electronic medical record system.

### Participants

The source population for this study comprised all Danish residents 18 years and older. We excluded people with existing diagnoses of cardiovascular disease, diabetes, or arthritis other than knee or hip osteoarthritis (e.g., rheumatoid arthritis, gout), as well as people who had undergone knee or hip joint surgery for reasons other than osteoarthritis. The exposed population comprised all adults with prevalent or incident knee osteoarthritis, hip osteoarthritis, or both knee and hip osteoarthritis.

Exposure status was first determined at baseline; we included all people with prevalent osteoarthritis who met our selection criteria. Control participants without knee or hip osteoarthritis and meeting our selection criteria were individually matched 5:1 on a set of potential confounders: age, sex, and level of education.

In accordance with Danish law governing the analysis of public registry data, the current study did not require ethical approval, and informed consent was not required. The study protocol was approved by the Danish Data Protection Agency (19/35402).

### Variables

#### Physician-diagnosed osteoarthritis exposures.

We identified exposures by relevant ICD-10 codes for knee and hip osteoarthritis. All participants with a diagnostic code indicative of hip (M16.0, M16.1, M16.2, M16.3, M16.4, M16.5, M16.6, M16.7, M16.9) or knee (M17.0, M17.1, M17.2, M17.3, M17.4, M17.5, M17.9) osteoarthritis in the Danish National Patient Register were assigned to the exposure group.

#### Cardiovascular disease outcomes.

Cardiovascular disease outcomes were identified by ICD-10 codes indicating angina pectoris, myocardial infarction, ischaemic heart disease, heart failure, atherosclerosis, stroke, and related conditions. Please refer to S1 Table for all ICD-10 codes used to define cardiovascular disease.

We undertook a planned sensitivity analysis using an alternative definition of cardiovascular disease to identify the potential for bias owing to outcome misclassification. The alternative outcome included a broader set of ICD-10 codes comprising a more comprehensive range of cardiovascular disease-related diagnoses (e.g., cardiomyopathy, myocarditis, atrial fibrillation). The complete list of the alternative ICD-10 codes is reported in S2 Table.

#### Potential confounders.

**Fig 1:**
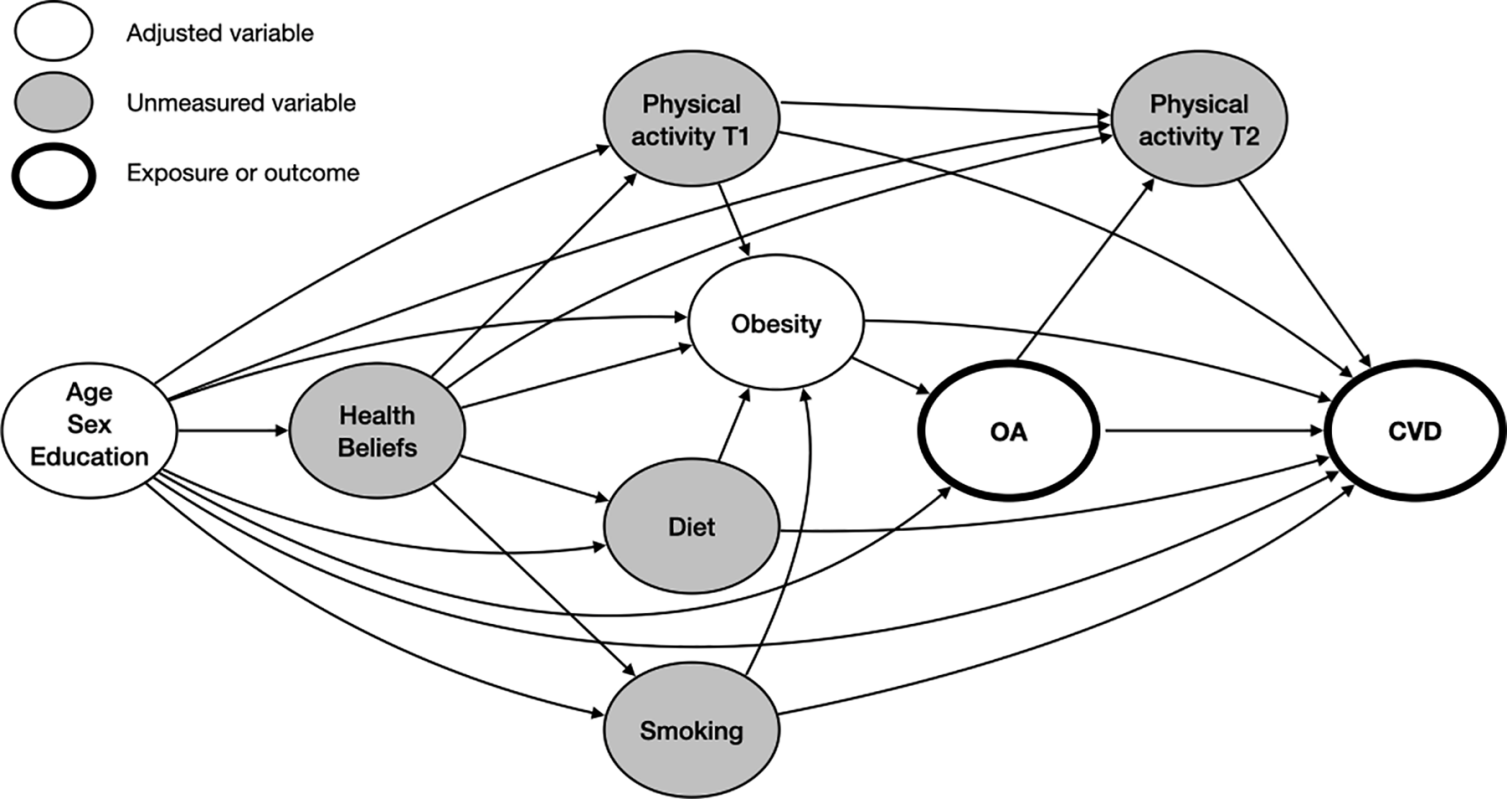
Directed acyclic graph demonstrating the relations between measured and unmeasured confounding variables, osteoarthritis, and cardiovascular disease.

To illustrate our assumptions about confounding and avoid inappropriate model adjustment, we constructed a directed acyclic graph with DAGitty v3.0 software (Fig 1).[25] Accounting for measured and unmeasured variables, the graph identified age, sex, education, and obesity as the minimally sufficient adjustment set to account for confounding in the relation between osteoarthritis and cardiovascular disease. The software systematically identifies adjustment sets based on causal inference theory to account for potential biases in exposure-outcome effects. Specific considerations include identifying the smallest set of variables that block all backdoor paths between the exposure and the outcome while avoiding conditioning on mediators or colliders.[26, 27]

Physical activity T1 represents the physical activity behaviour prior to the onset of osteoarthritis.

Physical activity T2 represents the physical activity behaviour subsequent to the development of osteoarthritis. OA =  osteoarthritis; CVD =  cardiovascular disease.

Data on baseline age, sex, and level of education were obtained from the Danish National Patient Register. We categorised age as 55 years or younger, 56–70 years, or greater than 70 years, owing to its non-linear association with cardiovascular disease risk. Categories for the level of education were high school or less, some university or trade school, or undergraduate or graduate university degree. Relevant ICD-10 codes identified participants who developed obesity during the study period: E66.0, E66.1, E66.2, E66.8, and E66.9.

### Statistical Methods

We described continuous variables with means and standard deviations (SD) and categorical variables with counts and percentages. The study outcome was time until cardiovascular disease diagnosis. Using cumulative incidence curves, we visually depicted differences in the cumulative incidence of cardiovascular disease (expressed as a proportion) between participants with and without osteoarthritis. To estimate the effects of hip or knee osteoarthritis on cardiovascular disease, we constructed sex-stratified multivariable Cox proportional hazard models. These models controlled for *a priori* identified confounders, including the matching variables of age, sex, and educational status, as well as obesity diagnosis. All model outcomes were censored on 31 December 2015. We plotted Schoenfeld residuals to evaluate the assumption of proportional hazards and detected no violations.[28] Model results were reported with hazard ratios (HR) and 95% confidence intervals.

We undertook two sensitivity analyses. First, we repeated our modelling strategy using a broader definition of cardiovascular disease. Second, we used E-value methodology for HR with commonly occurring outcomes to assess the robustness of model results to unmeasured confounding.[29] E-values estimate the minimum strength of association on the risk ratio scale that an unmeasured confounder would need to have with both the exposure and the outcome, conditional on the measured covariates, to fully explain away an exposure-outcome association. All analyses were performed in R (R Core Team, 2020) with statistical significance set to a 2-sided P value of.05.

## Results

### Participants and descriptive data

**Fig 2:**
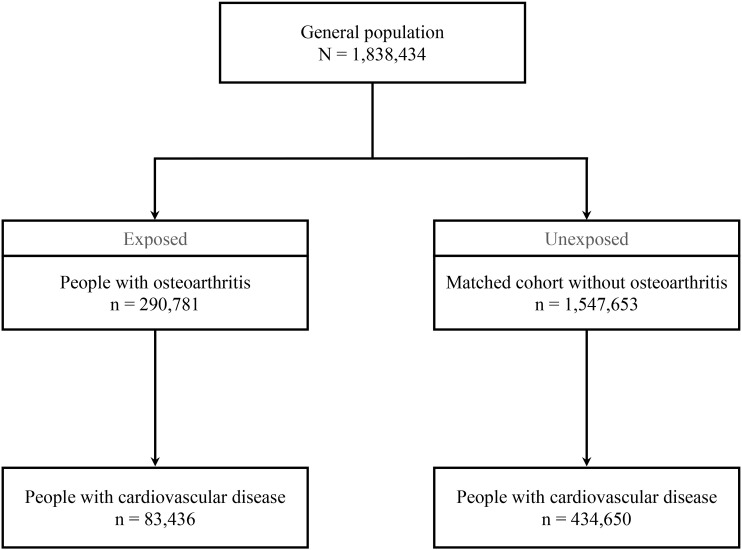
Participant flow chart. Main results.

We considered data from 1,838,434 adult residents of Denmark: 290,781 people with knee or hip osteoarthritis, and 1,547,653 age-, sex-, and education-matched controls (Table 1, Fig 2). In total, 19,561 people (6.7%) in the osteoarthritis group and 58,041 people (3.8%) in the control group were diagnosed with obesity.

**Table 1:** Description of the study sample by exposure status and sex (N =  1,838,434)^a^.

Variables	People with osteoarthritis (n = 290,781)		People without osteoarthritis (n = 1,547,653)	
	Women (n = 171,340)	Men (n = 119,441)	Women (n = 857,380)	Men (n = 690,273)
Age				
≤ 55 years 56 to 70 years > 70 years	20,815 (12.2%) 50,958 (29.7%) 99,567 (58.1%)	23,514 (19.7%) 43,071 (36.1%) 52,856 (44.3%)	95,010 (11.1%) 296,327 (34.6%) 466,043 (54.4%)	106,288 (15.4%) 264,333 (38.3%) 319,652 (46.3%)
Education level				
High school or less Trade education University/postgraduate	97,325 (56.8%) 37,560 (21.9%) 36,455 (21.3%)	51,335 (43.0%) 50,345 (42.2%) 17,761 (14.9%)	485,178 (56.6%) 194,593 (22.7%) 177,609 (20.7%)	304,275 (44.1%) 291,096 (42.2%) 94,902 (13.8%)
Diagnosed obesity	13,870 (8.1%)	5,691 (3.8%)	38,916 (4.5%)	19,125 (2.8%)

^a^ Values are counts (percentage).

#### Effects of osteoarthritis on cardiovascular disease in women and men.

**Fig 3:**
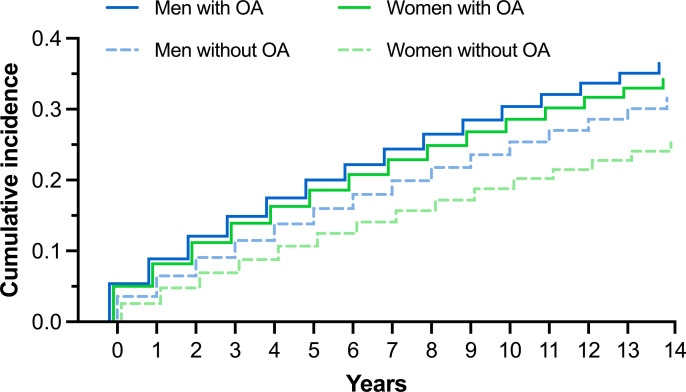
Cumulative incidence curves of cardiovascular disease for men and women with and without hip or knee osteoarthritis.

Figure 3 shows the cumulative incidence of cardiovascular disease for women and men with and without knee or hip osteoarthritis. Women with knee or hip osteoarthritis had a 44% increased hazard of subsequent cardiovascular disease (HR [95% CI] =  1.44 [1.43 to 1.46]) compared to women without osteoarthritis, after controlling for age, education, and diagnosed obesity. Men with knee or hip osteoarthritis had a 24% increased hazard of subsequent cardiovascular disease (HR[95% CI] =  1.24 [1.23 to 1.26]) compared to men without osteoarthritis, after controlling for age, education, and obesity diagnosis.

### Sensitivity analyses

When applying our more comprehensive definition of cardiovascular disease, the models demonstrated similar, albeit slightly higher, estimates for the association between osteoarthritis and cardiovascular disease. Women with knee or hip osteoarthritis had a 48% increased hazard of subsequent cardiovascular disease (HR [95% CI] =  1.48 [1.46 to 1.49]) compared to women without osteoarthritis, after controlling for age, education, and obesity diagnosis. Men with knee or hip osteoarthritis had a 30% increased hazard of subsequent cardiovascular disease (HR [95% CI] =  1.30 [1.28 to 1.31]) compared to men without osteoarthritis, after controlling for age, education, and obesity diagnosis.

In women, the calculated E-values for the point estimate and lower bound of the confidence interval of the estimate were 1.89 and 1.88, respectively. This means that with an observed HR (95% CI) =  1.44 (1.46 to 1.49), an unmeasured confounder associated with both osteoarthritis and cardiovascular disease, equivalent to a risk ratio of 1.89 each, above and beyond the measured confounders, could explain away the estimate, but weaker confounding could not. Further, an unmeasured confounder associated with exposure and outcome by a risk ratio of 1.88, above and beyond the measured confounders, could shift the CI to include the null, but weaker confounding could not. The corresponding E-values for men were 1.59 for the point estimate and 1.58 for the lower bound of the confidence interval. To place these results in context, the HR (95% CI) for diagnosed obesity in our model was 1.48 (1.46 to 1.50) for women and 2.16 (2.12 to 2.20) for men. This means that substantial unmeasured confounding, similar in magnitude to obesity, would be required to explain away the effect of osteoarthritis on cardiovascular disease.

## Discussion

The results of this study provide precise estimates of the adverse impact of knee and hip osteoarthritis on the risk of developing cardiovascular disease. After controlling for age, sex, education, and obesity, people with physician-diagnosed osteoarthritis were at increased risk of cardiovascular disease compared to age-, sex-, and education-matched control participants. The apparent effect of osteoarthritis on cardiovascular disease was stronger in women than in men. These results were confirmed by a sensitivity analysis evaluating the impacts of outcome misclassification and unmeasured confounding.

The current results address relevant knowledge gaps reported by a recent systematic review that highlighted the lack of structural strategies for confounder selection and assessment of unmeasured confounding in previous studies.[14] However, the current estimates tended to be greater than those reported in previous studies investigating the relationships between osteoarthritis and cardiovascular disease or cardiovascular mortality. For example, a previous prospective study reported an increased risk of hospitalization for cardiovascular disease among patients with osteoarthritis that ranged from risk ratio =  1.08 to 1.26 depending on age and sex.[30] Similarly, a longitudinal study of women reported an increased risk of cardiovascular disease-specific mortality in participants with degenerative changes on hip x-ray compared to controls (HR =  1.24),[31] while another cohort study reported HR from 1.07 to 1.29 between hip and knee osteoarthritis and cardiovascular disease diagnosis.[32]

A potential explanation for the discordancy in results may include modelling decisions around covariate selection. As discussed, the mechanisms that could explain the effect of osteoarthritis on cardiovascular disease include the impacts of joint pain on health-related physical activity. Since insufficient physical activity impacts many cardiovascular disease risk factors, including hypertension, hyperlipidemia, and diabetes mellitus,[33] their role in models attempting to estimate the effect of osteoarthritis on cardiovascular disease requires careful consideration. Previous analyses adjusting for these risk factors may have inadvertently controlled for variables along the causal pathway between osteoarthritis and cardiovascular disease. This practice would be expected to bias estimates toward the null (overadjustment bias), underscoring the benefit of structural approaches to confounder selection.[34]

### Clinical implications

These results suggest that clinicians should consider osteoarthritis as a risk factor for cardiovascular disease, particularly among women. This knowledge may inform clinical decision-making for patients with osteoarthritis as therapy selection can impact a patient’s risk for adverse cardiovascular events. The primary treatment goals for patients with osteoarthritis are to control pain and improve function. Treatment options include non-pharmacological and pharmacological therapies, as well as surgery. Exercise therapies such as walking, strengthening, and neuromuscular and aquatic training are recommended for all patients with hip or knee osteoarthritis.[35] Exercise and dietary modification can reduce pain, improve function, and promote weight loss, a key goal for patients with osteoarthritis living with overweight or obesity. Importantly, these non-pharmacological therapies are also effective for preventing and treating cardiovascular disease, a distinct advantage over other therapeutic options.

Clinicians who prescribe medications for osteoarthritis pain face complex decision-making concerning effectiveness and safety.[36] NSAIDs are commonly prescribed for patients with osteoarthritis,[37] with effectiveness comparable to exercise for patients with hip[38] or knee[39] osteoarthritis. However, effect estimates vary widely, and NSAIDs with greater evidence of effectiveness for osteoarthritis have been withdrawn from the market in many countries.[40] Moreover, unlike exercise and physical activity, which reduce the risk of cardiovascular disease, NSAIDs can increase the risk of myocardial infarction, stroke, and cardiovascular death.[41]

Patients with persistent osteoarthritis disability who do not benefit from non-operative therapies may be candidates for total joint replacement. The outcomes experienced by patients who undergo joint replacement surgery for osteoarthritis are generally considered good, with approximately 90% of total hip replacement recipients and 80% of total knee replacement recipients reporting favourable results.[42–44] Yet, despite improvements in pain, disability, and physical capacity, there is little change in health-related physical activity behaviour following joint replacement surgery.[45, 46] This reality should not be surprising; physical activity is a complex multidimensional behaviour[47] that is unlikely to change without professional intervention and ongoing support. This suggests that the full benefits of joint replacement surgery are not being realised and that behaviour-change interventions are warranted for patients with osteoarthritis.

### Research implications

The current study may inform future research aimed at understanding the relationship between osteoarthritis and cardiovascular disease. Research priorities should include identifying the mechanisms by which osteoarthritis may impact the development of cardiovascular disease. As discussed, current hypotheses include reduced health-related physical activity, use of NSAIDs, and persistent inflammation. This knowledge will be key to patient management as it can identify potentially modifiable targets for intervention. Contemporary approaches to mediation analysis are recommended to identify causal pathways,[48] while randomised trials identify the effects of interventions. For example, suppose causal mediation models were to identify decreased physical activity as a key mechanism for the development of cardiovascular disease in people living with osteoarthritis. In that case, randomised trials could identify the most effective interventions to improve physical activity behaviour.

### Strengths and limitations

These study results should be considered in light of several strengths and limitations. This was a national, 15-year longitudinal study yielding precise population-based effect estimates that accounted for multiple sources of confounding through participant matching and statistical adjustment. We selected confounders and illustrated our assumptions regarding the nature of the relations between potential confounders, physician-diagnosed osteoarthritis, and cardiovascular outcomes with a structural identification approach. The study results were confirmed by sensitivity analyses evaluating outcome misclassification and unmeasured confounding.

A primary assumption inherent to effect estimation with observational data is exchangeability through the complete control of confounding.[49] The current study results should be interpreted in the context of potential unmeasured and residual confounding. We selected confounders by constructing a directed acyclic graph based on existing evidence. This had two main advantages: to transparently communicate the assumptions inherent to our analyses and to help avoid inappropriate statistical adjustment. However, the accuracy of our theoretical model is not established, and incorrect specification of these associations is a potential source of bias. Although we accounted for multiple sources of confounding through the matched design and statistical adjustment, the participants with and without osteoarthritis may have differed in relevant ways. Nevertheless, our sensitivity analysis provides some measure of confidence that unmeasured confounding does not explain the observed effect of osteoarthritis on cardiovascular disease.

We evaluated the impact of competing risks with the cumulative incidence function, an appropriate non-parametric approach that provides an easy-to-interpret visualization of time-to-event results. More complex alternatives, such as Fine-Gray models, offer additional advantages, including the incorporation of multiple covariates. However, these models are intended for prognostic analyses [50] and may be prone to bias when multiple competing events are present.[51] The ideal approach to dealing with competing risks when modelling the effect of osteoarthritis on cardiovascular disease should be explored in future studies. The 10-year enrolment period may have introduced time-dependent bias due to factors such as evolving diagnostic criteria or changes in population health behaviours. Further, we were unable to evaluate for time-varying confounding, which may have oversimplified the relations between physical activity, obesity, and osteoarthritis. For example, while obesity is a risk factor for osteoarthritis, it can also result from insufficient physical activity owing to osteoarthritis-related pain. Future research should prioritize investigating these time-varying relationships.

Finally, misclassification is a challenge inherent to observational studies. We controlled for the effects of diagnosed obesity as a common cause of osteoarthritis and cardiovascular disease. However, diagnosed obesity can misclassify people with excessive body mass who remain undiagnosed. Consequently, our obesity measure may represent an overly conservative obesity estimate in this population and is a potential source of residual confounding. Similarly, our sensitivity analysis for outcome misclassification yielded larger effect estimates, indicating that some people in the primary model may have been incorrectly specified. Consequently, the primary analyses may represent overly conservative estimates of the effect of osteoarthritis on cardiovascular disease.

## Conclusion

Women and men with physician-diagnosed hip or knee osteoarthritis were at increased risk of cardiovascular disease compared to age-, sex-, and education-matched participants without osteoarthritis. The apparent effect of osteoarthritis on cardiovascular disease was stronger in women. Clinicians who care for patients with osteoarthritis should be aware of cardiovascular disease risk when selecting therapies and consider behavioural approaches to improving health-related physical activity behaviour in this population. Future research is needed to better define the mechanisms by which osteoarthritis impacts cardiovascular disease and to help identify effective interventions targeting these mechanisms.

## Supporting information

S1 Table ICD-10 codes used to classify cardiovascular disease in primary analyses. (PDF)

S2 Table Expanded list of ICD-10 codes used to classify cardiovascular disease in sensitivity analyses. (PDF)

## Abbreviations and acronyms:

NSAIDs: non-steroidal anti-inflammatory drugs
ICD-10: international classification of disease version 10
HR: hazard ratio.
