# Supplementary material for: Knee and hip osteoarthritis increase the risk of cardiovascular disease: A national registry-based longitudinal cohort study
Source: PLoS One. 2025 Apr 15;20(4):e0321290. doi: 10.1371/journal.pone.0321290 (PMC11999113; doi:10.1371/journal.pone.0321290)
Supplement: S1 Table — (PDF) [file pone.0321290.s001.pdf]

Supplementary Table 1. ICD-10 codes used to classify cardiovascular disease in primary analyses.

ICD-10 code	Description
I20	Angina pectoris
I21	Acute myocardial infarction
I22	Subsequent myocardial infarction
I23	Certain current complications following acute myocardial infarction
I25	Chronic ischaemic heart disease
I50	Heart failure
I51	Complications and ill-defined descriptions of heart disease
I52	Other heart disorders in diseases classified elsewhere
I60	Subarachnoid haemorrhage
I61	Intracerebral haemorrhage
I62	Other nontraumatic intracranial haemorrhage
I63	Cerebral infarction
I64	Stroke, not specified as haemorrhage or infarction
I65	Occlusion and stenosis of precerebral arteries, not resulting in cerebral infarction
I66	Occlusion and stenosis of cerebral arteries, not resulting in cerebral infarction
I67	Other cerebrovascular diseases
I68	Cerebrovascular disorders in diseases classified elsewhere
I69	Sequelae of cerebrovascular disease
I70	Atherosclerosis

ICD-10, International Classification of Diseases, Tenth Revision
