# Supplementary material for: Knee and hip osteoarthritis increase the risk of cardiovascular disease: A national registry-based longitudinal cohort study
Source: PLoS One. 2025 Apr 15;20(4):e0321290. doi: 10.1371/journal.pone.0321290 (PMC11999113; doi:10.1371/journal.pone.0321290)
Supplement: S2 Table — (PDF) [file pone.0321290.s002.pdf]

Supplementary Table 2. Expanded list of ICD-10 codes used to classify cardiovascular disease in sensitivity analyses.

ICD-10 code	Description
I20	Angina pectoris
I21	Acute myocardial infarction
I22	Subsequent myocardial infarction
I23	Certain current complications following acute myocardial infarction
I24	Other acute ischaemic heart diseases
I25	Chronic ischaemic heart disease
I26	Pulmonary embolism
I27	Other pulmonary heart diseases
I28	Other diseases of pulmonary vessels
I30	Acute pericarditis
I31	Other diseases of pericardium
I32	Pericarditis in diseases classified elsewhere
I33	Acute and subacute endocarditis
I34	Nonrheumatic mitral valve disorders
I35	Nonrheumatic aortic valve disorders
I36	Nonrheumatic tricuspid valve disorders
I37	Pulmonary valve disorders
I38	Endocarditis, valve unspecified
I39	Endocarditis and heart valve disorders in diseases classified elsewhere
I40	Acute myocarditis
I41	Myocarditis in diseases classified elsewhere
I42	Cardiomyopathy
I43	Cardiomyopathy in diseases classified elsewhere
I44	Atrioventricular and left bundle-branch block
I45	Other conduction disorders
I46	Cardiac arrest
I47	Paroxysmal tachycardia
I48	Atrial fibrillation and flutter
I49	Other cardiac arrhythmias
I50	Heart failure
I51	Complications and ill-defined descriptions of heart disease
I52	Other heart disorders in diseases classified elsewhere
I60	Subarachnoid haemorrhage
I61	Intracerebral haemorrhage
I62	Other nontraumatic intracranial haemorrhage
I63	Cerebral infarction

I64	Stroke, not specified as haemorrhage or infarction
I65	Occlusion and stenosis of precerebral arteries, not resulting in cerebral infarction
I66	Occlusion and stenosis of cerebral arteries, not resulting in cerebral infarction
I67	Other cerebrovascular diseases
I68	Cerebrovascular disorders in diseases classified elsewhere
I69	Sequelae of cerebrovascular disease
I70	Atherosclerosis
I71	Aortic aneurysm and dissection
I72	Other aneurysm and dissection
I73	Other peripheral vascular diseases
I74	Arterial embolism and thrombosis
I77	Other disorders of arteries and arterioles
I78	Diseases of capillaries
I79	Disorders of arteries, arterioles and capillaries in diseases classified elsewhere

ICD-10, International Classification of Diseases, Tenth Revision
